# Testing the Hayek hypothesis: Recent theoretical and experimental evidence

**DOI:** 10.1371/journal.pone.0270489

**Published:** 2022-07-14

**Authors:** Omar Al-Ubaydli, Peter Boettke, Brian C. Albrecht

**Affiliations:** 1 Bahrain Center for Strategic, International and Energy Studies and Department of Economics and Mercatus Center, George Mason University, Fairfax, VA, United States of America; 2 Department of Economics and Mercatus Center, George Mason University, Fairfax, VA, United States of America; 3 International Center for Law and Economics and Coles College of Business, Kennesaw State University, Kennesaw, GA, United States of America; Universitat Jaume I, SPAIN

## Abstract

Economists well understand that the work of Friedrich Hayek contains important theoretical insights. It is less often acknowledged that his work contains testable predictions about the nature of market processes. Vernon Smith termed the most important one the ‘Hayek hypothesis’: that gains from trade can be realized in the presence of diffuse, decentralized information, and in the absence of price-taking behavior and centralized market direction. Vernon Smith tested this prediction by surveying data on laboratory experimental markets and found strong support. We extend Smith’s work first by showing how subsequent theoretical advances provide a theoretical foundation for the Hayek Hypothesis. We then test the hypothesis using recent field experimental market data. Using field experiments allows us to test several other predictions from Hayek, such as that market experience increases the realized gains from trade. Generally speaking, we find support for Hayek’s theories.

## 1. Introduction

Friedrich Hayek’s writings on the market process, in general, and his most famous “The Use of Knowledge in Society” [1], in particular, provide several testable predictions about the market process. The most important prediction, which Vernon Smith [2] called the ‘Hayek hypothesis’, is that the gains from trade can be realized in the presence of diffuse, decentralized information, and in the absence of price-taking behavior and centralized market direction (see [3] for a more follow-up test). Critically, as Smith noted, these predictions are sometimes at odds with the standard interpretation of the competitive, Walrasian model.

To test Hayek’s theories of the market process, Smith [2] reviewed the extensive laboratory evidence and found it to be consistent with the Hayek hypothesis. Smith went on to pose the following question: “… does this mean that it will do comparably well in the ‘field’ environment of the economy? … few such field experiments have been attempted,” [2: p177]. Forty years later, we no longer need to speculate. Using the recent surge of field experimental research, this paper picks up where Smith left off and assesses how well Hayek’s theories travel in the field.

The advantages of laboratory experimental data over naturally occurring data are well-documented [4]. In the context of market experiments, the most important is the control that the laboratory affords the investigator, permitting him to induce demand and supply, and to have full knowledge of the predicted equilibrium. Field experiments complement their laboratory counterparts by, among other things, allowing the investigator to observe more diverse contexts and to limit the difficulties arising from inexperienced experimental subjects [5, 6]. In particular, the field experiments can more closely approximate naturally occurring markets compared to using students in a lab, which is the ultimate object of focus for Hayek, and us.

We find that Hayek’s theories are well supported by the field experimental data overall. However, we also find that there are important exceptions that open the door to refinements of Hayek’s theories. For example, in certain environments, the presence of experienced entrepreneurs and the dissemination of price information can hinder a market’s ability to realize the gains from trade.

The remainder of this paper is organized as follows. Section 2 is a brief overview of experimental methods. Section 3 first builds on theoretical work done since Smith to provide a framework to understand Hayek’s claims. Then Section 3 states Hayek’s testable theories. Section 4 reviews the field experimental evidence on these theories. Section 5 concludes.

## 2. Experimental methods

To fully comprehend the implications of field experimental data for Hayek’s theories, we need to lay out the exact advantages implied by experimental methods in general, as well as the relevant advantages of field experiments. For a full discussion of laboratory experiments, see [7, 8] and for field experiments, see [5].

### 2.1. Laboratory experiments

Hayek’s market theories are more easily tested if the investigator knows the demand and supply schedules. In the absence of massive (and untestable) structural assumptions, this information is impossible to obtain from naturally occurring data. The laboratory allows the investigator to precisely induce the demand and supply schedules [2, 9]. The investigator can also control (partially or completely):

- The information available to market participants
- The communication permissible between market participants
- The contracts available to market participants
- The horizons of interaction permissible between market participants

### 2.2. Field experiments

There are three types of field experiment which lie within a larger taxonomy (see [5])

- **Conventional lab experiment**: a standard subject pool of students, abstract framing, and an imposed set of rules
- **Artefactual field experiment**: same as conventional laboratory experiments but with a non-standard subject pool, e.g., having general members of the population participate in a laboratory market
- **Framed field experiment**: same as an artefactual field experiment but with a field context in either the commodity, task, or information that the subjects can use, e.g., having professional traders participate in a laboratory market that functions similarly to the market in which they normally operate
- **Natural field experiment**: same as a framed field experiment but where the environment is the real environment where the subjects undertake these tasks, and where the subjects are not aware that they are in an experiment, e.g., performing an experiment at the New York Stock Exchange without the knowledge of the traders

In the case of markets with induced demand and supply schedules, natural field experiments are impossible; the fact that the demand and supply schedules are induced means they cannot be naturally occurring. Compared to the remaining field experiments, laboratory experiments potentially suffer from the following drawback [4–6]: subjects may be inexperienced in the task and self-selection into the experiment may bias results. For example, the outcomes of a market may depend upon the inept traders having been whittled out, which is much less likely to have occurred in a laboratory experiment. This may be particularly important in the context of Hayek’s theories.

An additional advantage of field experiments is the ability to consider longer-term issues such as reputation. When using students as subjects in an unnatural environment, reputational horizons rarely extend into months or years, in contrast to using subjects drawn from the natural environment being studied; field experiments extend the domain of analysis. These extensions are important to understanding Hayek’s predictions about the market process, as we will show below.

Even setting aside these potential advantages of field experiments, it is useful to extend the domain of inquiry to as many different markets as possible purely as a standard act of robustness-checking. Most likely, this is what Smith had in mind when he posed the question about the generalizability of the laboratory support for Hayek’s theory to field settings [2]. We very much regard laboratory experiments and field experiments as complements in the study of market processes.

## 3. Hayek’s testable theories

### 3.1. Framework

**Fig 1:**
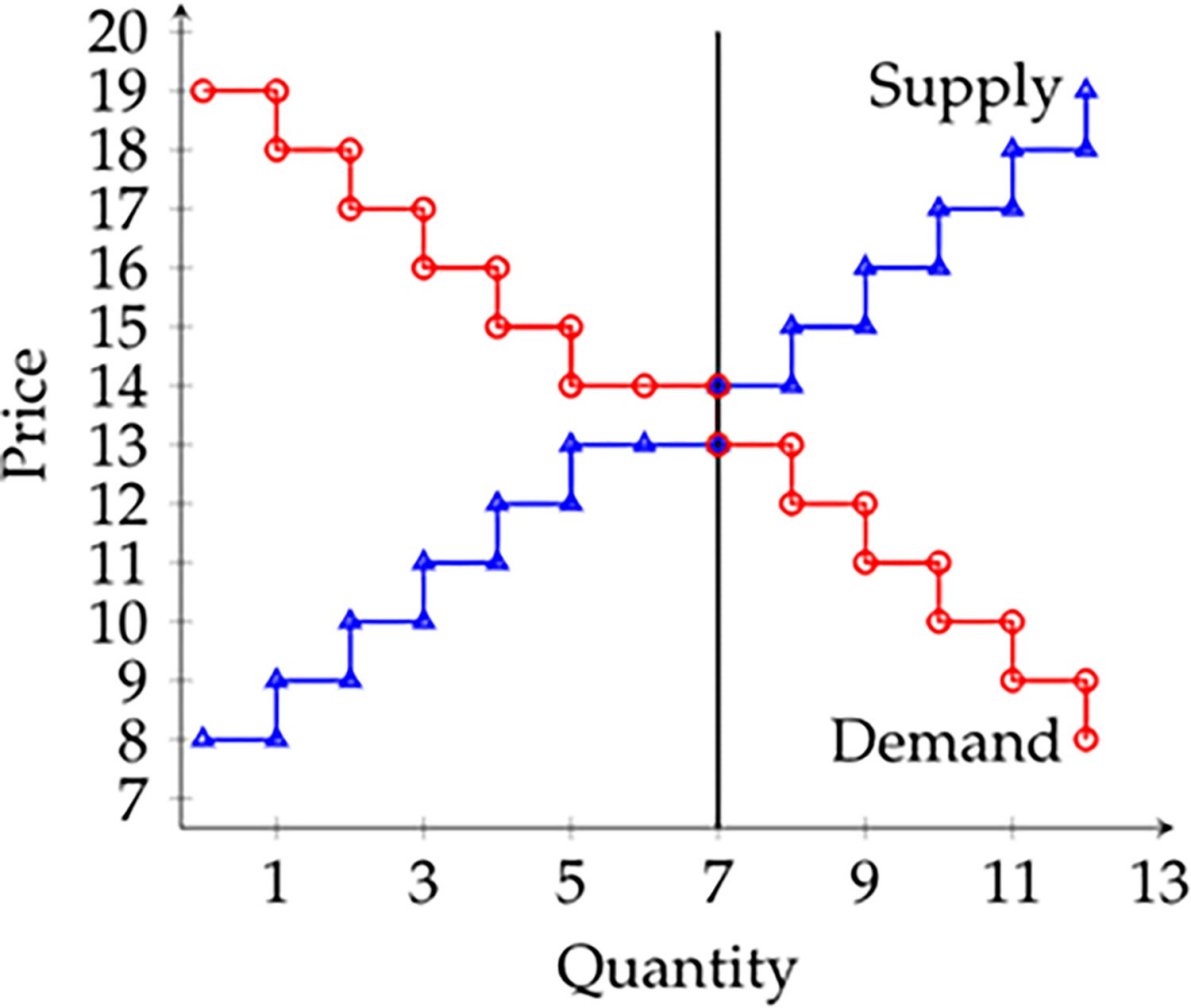
Values/costs in symmetric markets in [11].

Hayek’s testable theories can be reduced to predictions about features of trading institutions that increase the likelihood of efficient trades occurring. Before getting to particular institutions, it will be helpful to describe a class of general equilibrium models that are used in field experiments. We follow the model in [10], which is simple but general. There are two goods: ‘money’ and a traded good. Preferences are such that each individual’s demand curve for the traded good (in terms of ‘money’) is a step function, as in Fig 1 below from [11]. In the experiment, units of utility are exchanged for real money at some fixed rate. Utility is transferrable. This framework is employed in conjunction with a variety of trading institutions, including Chamberlin markets [12] and double oral auctions [13].

Let I = B∪S be the set of players. We will call them buyers and sellers. For each i∈I, her preferences can be described by two k-vectors, $\left(r_{1}^{i},\ldots ,r_{k}^{i}\right)$ and $\left(q_{1}^{i},\ldots ,q_{k}^{i}\right)$, which are the reservation values and quantities for each reservation value. For each buyer, i∈B, reservation values decrease with quantity and for each seller i∈S the reservation values increase with quantity.

For a player i with parameters $\left(r_{j}^{i}\right)$ and $\left(q_{j}^{i}\right)$, denote the quantity of trades by $y^{i}\in (q_{j-1}^{i},q_{j}^{i}]$ for some 1≤j≤k. If we normalize the utility for zero trade to be zero, i.e., v^i^(0) = 0, and normalize $q_{0}^{i}=0$ then total utility of y^i^:

$$v^{i}(y^{i})=\sum_{l=1}^{j-1}r_{l}^{i}(q_{l}^{i}-q_{l-1}^{i})+r_{j}^{i}(y^{i}-q_{j-1}^{i}).$$

The total payoff to each individual will be the utility received from the traded good, v^i^(y^i^), minus the money paid or plus the money received.

Let ${V^{i}}^{2k}$ be the set of all utility functions with at most 2k parameters. Then an economy is defined by:

$$v\in V^{2k}=\underset{i}{\times }{V^{i}}^{2k}.$$

We can now differentiate classes of economies used within field experiments. If buyers and sellers can only trade one indivisible unit of the good, then $q_{1}^{i}\equiv 1$ and the number of parameters is k = 1. Call this class of economies V^1^. If buyers and sellers can also choose the quantity to trade, call this class of economies V^2^. One important difference for economies in V^2^ compared to V^1^ is that there are more gains from strategic misreporting. Instead of only being able to underreport their valuation, the players can also try to strategically manipulate the quantity they offer in order to gain more favorable trading terms. While these cases describe most experiments, the formal theorem below will also apply to the general class of environments, given by V^h^, where h = 1,2,4,6…. Note that since for any h<h′, V^h^⊂V^h′^, the theorem will apply to economies with fewer parameters.

On top of this physical environment, we can overlay the rules of a specific market institution and the related information structure. Traders interact by exchanging messages over time with other traders. The most important messages are typically offering trade prices and accepting trade offers. Other messages correspond to the general bargaining process. Specific markets may have rules about the messages that can be exchanged; for example, in an English auction, a central (non-trading) auctioneer is the only person who calls out prices, and the traders are restricted to expressing a willingness to trade at the prevailing price.

Even though Hayek was not particularly concerned with Walrasian equilibria, it is helpful to start there as a baseline and because of the well-known connection between Walrasian equilibria and the realized gains from trade, i.e., The First Welfare Theorem.

A competitive equilibrium, or more precisely, a *Walrasian equilibrium* for v∈V^k^ consists of a price p and quantities traded (y^i^), such that:

1. (Agents maximize utility) for each i, $v^{i}(y^{i})-py^{i}\geq v^{i}(x^{i})-px^{i}$, where $0\leq x^{i}\leq q_{k}^{i}$ if i∈B and $0\geq x^{i}\geq q_{k}^{i}$ if i∈S, and
2. (Markets clear) ∑y^i^ = 0.

Denote the set of prices p and the set of allocations (y^i^) that form part of some Walrasian equilibrium by P(v) and Y(v), respectively. With quasi-linear preferences, like we are dealing with here, it is well known that the set of pairs of prices and allocation that make a Walrasian equilibrium for an economy v is P(v)×Y(v).

Instead of a Walrasian mechanism, to more closely model the field experiments we want to consider a mechanism that 1) makes sense with small numbers of players, 2) allows players to choose prices, and 3) depends on decentralized bids and offers.

For simplicity in the model, even if market institutions are different in the actual experiments, we can think of trading as operating through a clearinghouse that clears supply and demand. An individual with preferences given by $v^{i}=((r_{j}^{i}),(q_{j}^{i}))\in {V^{i}}^{h}$ can submit any bid/offer that is feasible. A feasible bid offer is $w^{i}=(({\hat{r}}_{j}^{i}),({\hat{q}}_{j}^{i})),$ such that $w^{i}\in {V^{i}}^{h}$ and $\left|{\hat{q}}_{j}^{i}|\leq |q_{j}^{i}\right|$. The quantity restriction rules out the ability to offers that exceed one’s capacity, but it allows the individual to withhold goods, say in hopes of gaining favorable trading terms.

To allow the possibility of strategic behavior, one can consider Nash equilibrium. Let w^−i^ be the set of bid/offers with player i removed. A set of bids/offers w∈V^h^ is a Nash equilibrium for v∈V^h^ if for each i, $w^{i}\in {V^{i}}^{h}(v^{i})$, and for all ${\hat{w}}^{i}\in {V^{i}}^{h}(v^{i})$,

$$v^{i}[y^{i}(w)]-p(w)y^{i}(w)\geq v^{i}[y^{i}(w^{-i},{\hat{w}}^{{}^{i}})]-p(w^{-i},{\hat{w}}^{{}^{i}})y^{i}(w^{-i},{\hat{w}}^{{}^{i}}).$$

There is always a trivial Nash equilibrium where everyone announces $q_{k}^{i}=0$ and no trade occurs. We will only focus on the set of equilibria with positive trade.

Right around the time of [2], important papers by Dubey, Simon, and Benassy proved the key link between Nash equilibria and Walrasian equilibria (and by extension efficient allocations) [14–16]. The key condition is that both sides of the market have active competition. Formally, for any v∈V^h^ for which (y^i^)∈Y(v), it must be the case that #{i: y^i^≠0, i∈B}≥2 and #{i: y^i^≠0, i∈S}≥2. In words, a Nash equilibrium involves active competition if there are at least two buyers and two sellers trading in equilibrium. Following [10], we can state the following theorem:

**Dubey-Simon-Benassy Theorem:** For any economy v∈V^h^ permitting active competition from both sides of the market, the set of positive trade Nash equilibrium outcomes is equal to the set of Walrasian equilibria.

Contrary to the common belief that competitive outcomes require many players, the Dubey-Simon-Benassy Theorem shows that two on each side is enough. The idea that *price* competition between two players can generate competitive outcomes goes back before Hayek to Bertrand. Following up on Demsetz’s idea of competition “for the field,” recent studies have showed how a monopoly active trader on each side of the market is compatible with competition when there are non-active traders outside the field that will enter if either side tries to exert any market power [17]. See [18–20] for recent examples.

The above framework and theorem should be seen as a baseline lens through which to interpret the more complicated hypotheses in Hayek that are more difficult to formalize. For example, traders usually differ in their information sets depending on the market institution. First and foremost, they are (typically) imperfectly/incompletely informed about other traders’ preferences and production sets. In some markets, an individual trader may be better-informed about the preferences of another trader over a specific unit than the latter trader himself, e.g., the lemons problem [21]. Traders can also vary in their knowledge of previous messages exchanged by other traders (e.g., offers and trade prices), both within and beyond the current trading session.

In response to the imperfect information, traders form beliefs; we do not restrict these beliefs to being rational; thus, we allow for the possibility of unknown unknowns: traders not even being aware of the possibility that certain features of the market exist, as in [19]. Upon learning their preferences and forming their beliefs, traders interact using messages subject to the rules of the institution. Their choices need not be strictly rational; they can be near-rational [22] or they may involve heuristics.

This framework is useful because if we operationalize Hayek’s theories, the principal dependent variable is the efficiency of an allocation, and the principal explanatory variables are features of the trading institution and trader attributes.

### 3.2. Hayek’s theories

In contrast to the general framework above, Hayek argues *against* a model of behavior where agents’ preferences are determined separately from market processes; he believes that the process of competition is central to how agents assess how much they value commodities [23, 24]. At face value, this poses difficulties for inducing preferences [9] in an experiment.

To overcome this [2], relaxed some of the Hayekian assumptions, noting that much of what Hayek proposed about markets applies in principle even if preferences can be induced. We follow [2] and thus the hypotheses that we present and test can be considered as (Vernon) Smithian interpretations of Hayek.

### 3.3. Hypothesis 1: The Hayek hypothesis

Smith remarks that the standard proposition of decentralized market theory is that the gains from trade will be exhausted; further, prices will converge to the competitive equilibrium implied by the intersection of demand and supply [2]. The divergence between Hayek and neoclassical scholars arises when one considers the conditions necessary for these outcomes, and the precise mechanism by which the competitive equilibrium is attained (see the section on alternative theories below).

As Smith has more recently stated, “Experiments constituted a substitute for the missing dynamic process analysis that had not been part of the standard equilibrium tool kit that had only focused only on what might be the equilibrium shadow cast ahead by any such process” [25]. Experimental economics in other words was providing a demonstration of what Hayek was talking about in terms of “knowledge-acquisition” and the dynamic adjustments that characterize the entrepreneurial market process.

Hayek had long argued that the preoccupation with the equilibrium state of affairs had clouded economic analysis. It is important to stress that Hayek never rejected equilibrium economics, but he wanted to shift the analytical focus of economists to the processes of knowledge-acquisition and communication by economic actors such that would bring about the equilibrium state of affairs.

Hayek coming out of the Viennese tradition of economics had always focused on the step-by-step, or process analysis, as opposed to either the Walrasian or Marshallian traditions of price theory (see, e.g., [26, 27: p17] argued that the goal of economic analysis should be to provide a causal explanation of the process in time which brings about the complex coordination of plans. Equilibrium analysis in this perspective is “significant only in so far as it is preparatory to this main task.” Economic problems result because of changing conditions, and the price system works “as a kind of machinery for registering change.” [1: p87] The structure of property rights and the constellation of prices within the market both incentivize and provide guiding signals to actors to coordinate their affairs; absent that context, the dispersed knowledge within the economy that resides in specific times and places will not be discovered, used, or communicated.

As Hayek stressed, he was far from denying that equilibrium analysis had a significant role to play in economic analysis. But a preoccupation with the equilibrium solution misled leading thinkers into ignoring the institutional context of choice and the social process of exchange and production. The market economy is an emergent order. Or, as James Buchanan [28] has put it in a classic paper extending Hayek’s analysis of the market order:

A market is not competitive by assumption or by construction. A market *becomes* competitive, and competitive rules *come to be* established as institutions emerge to place limits on individual behavior patterns. It is this *becoming* process, brought about by the continuous pressure of human behavior in exchange, that is the central part of our discipline, if we have one, not the dry rot of postulated perfection. A solution to a general-equilibrium set of equations is not predetermined by exogenously determined rules. A general solution, if there is one, *emerges* as a result of a whole network of evolving exchanges, bargains, trades, side payments, agreements, contracts which, finally at some point, ceases to renew itself. At each stage in this evolution toward solution there are *gains* to be made, there are exchanges possible, and this being true, the direction of movement is modified.

It is this evolution toward solution that was missing in the early formal theory. Vernon Smith [24: p80] has argued that one of the “important contributions of experimental economics has been to enable us to better understand the sequential dynamic process whereby markets achieve competitive equilibrium states.” It is this discovery process of equilibrium price formation that Hayek complained was ignored with the preoccupation with equilibrium modeling, and which Vernon Smith was able to illustrate in his market experiments.

**Hypothesis 1 (*The Hayek hypothesis*;[1])**: A large proportion of efficient rents will be realized even if:

- The number of traders *N* is small
- Traders can set prices
- Traders’ information about market conditions, including anything beyond their own preferences, is highly imperfect and incomplete
- Traders may or may not have rational expectations (or common knowledge) over market conditions
- There is no centralized market orchestrator

Our formal argument for parts a) and b) of Hypothesis 1 comes directly from the Dubey-Simon-Benassy Theorem. The theorem shows the tight link between the two ideas: when traders can set prices, competition will occur with a small number of traders.

Unfortunately, the remaining parts of Hypothesis 1 are not formally allowed in the equilibrium concept, which is an “as-if complete information” model. However, we agree with the interpretation in Friedman and Ostroy that “reliance on complete information is more terminological than substantive” [10: p32]. Furthermore, “Repetition, rather than complete information, permits conditioning on previous observation of market price. Because price conditioning is possible in the experiment but information is not complete, we call this an as-if complete information explanation” [10: p33]. They conclude “Our argument for the as-if complete information model is not based on logical or computational issues, but rather on the fact that in certain dynamic settings, the informational requirements for as-if complete information NE actually can be quite modest” [10: p48]). Their (and our) interpretation of the model allows for the people to start with incomplete information and non-rational expectations. It is through the market process that people discover more gains from trade. More recent work on market games, following up on [14–16], has explicitly incorporated incomplete information and non-rational expectations (e.g., [29]). Papers that focus on whether or not such an economy is competitive generally find support for the competition. See [30] for a recent example.

Returning to Hayek’s claims, we are left with the important question: how ‘large’? Unfortunately, Hayek makes no definitive claims. As we will see in the proceeding hypotheses, he does make predictions about factors that improve the functioning of markets, and so it would be inaccurate to use full efficiency as his prediction. Comparing markets to autarky is somewhat of a straw man. Hayek was more interested in comparing markets to government-mediated exchange systems, which is difficult to implement in a controlled experiment (field or otherwise). One possible way to test Hayek’s comparison more closely would be to require a central committee in the experiment to set “the market price” prices. One could then vary the information and the incentives of the central committee. A true government-mediated exchange system would rely on the committee’s own goodwill to get the price right for the sake of the market participants. In regards to the laboratory experimental literature, an informal and arbitrary heuristic that has emerged suggests that realized rents of 90% or above are considered ‘large’.

### 3.4. Hypothesis 2: Entrepreneurs and efficiency

Moving on to the next hypothesis, the prime mover in Hayek’s understanding of the competitive market process is the entrepreneur. Entrepreneurs are forever alert to opportunities for gains from trade and gains from innovation, and as such are the change agents within the economic system. “The practical problem is not whether a particular method would eventually lead to a hypothetical equilibrium, but which method will secure the more rapid and complete adjustment to the daily changing conditions in different places and different industries.” [23: p188] Acting on the opportunities for mutual gain that changing conditions present, the entrepreneur initiates the necessary adjustments. In examining the market economy, Hayek argued, it must never be forgotten that the opportunities for gains from trade and gains from innovation have “to be discovered, and to be discovered anew, sometimes almost day to day, by the entrepreneur.” [23: p196]. Hayek’s theory of the entrepreneurial market process received its most careful elaboration in the works of Israel M. Kirzner, most notably [31]. The focus in Kirzner is on the *entrepreneurial function* within the market economy, as opposed to a historical appreciation of any particular entrepreneur. In the Hayek-Kirzner presentation, the entrepreneur is the agent of change whose activity initiates the necessary relative price adjustments that are required to accommodate changing conditions, and lead to the coordinating of plans between buyers and sellers such that in the absence of any further change the market will clear. Rather than a Walrasian auctioneer reconciling the plans of economic actors in the market, a decentralized process of different actors being alert to opportunities for mutual gain drives the coordination of plans.

**Hypothesis 2 (Hayek [23, 24])**: Entrepreneurs have a positive causal effect on the realized proportion of efficient rents.

To test *Hypothesis 2*, we need to operationalize the concept of an entrepreneur. We argue that in a long-lived market, trader experience is a decent proxy for entrepreneurial ability. Experienced traders by definition have traded more than their inexperienced counterparts. Thus, for experience to proxy for Hayekian entrepreneurship, all that remains is for their higher volume of trades to reflect successful alertness to, and exploitation of, opportunities for mutual gain with other traders.

This latter point follows from the freedom of entry and exit in a market. If experienced traders were simply more experienced in making unprofitable (to themselves) trades, then under a mild form of rationality they would simply exit the market. (Even in the absence of mild rationality, a binding budget constraint would limit trading volume.) In contrast, if they were experienced in making trades that were profitable to themselves but unprofitable to others, then the market would be cannibalized and eventually cease to exist; hence our focus on long-lived markets. Thus, trading a lot more than others in a long-lived market must mean that you are particularly alert to opportunities for mutual gain and are therefore instrumental to the process by which efficient rents are realized.

To avoid the charge of tautology, let us clarify via a thought experiment. Imagine a market with entry and exit; as argued above, those adept at efficient trades will accumulate trading experience. Now suppose we freeze the market and exogenously alter the composition of experience in the market. If we decrease the volume of experienced traders–those who have been *in the past* successful at realizing efficient trades–then *from that point onwards* the rate at which efficient trades are realized will be diminished compared to a control group that experienced no intervention. The reason these people have lasted so long in the market is that they are good at spotting opportunities, and the market will suffer in their absence.

Clearly, experience is not an intrinsic component of the definition of the Hayekian entrepreneur. People who are particularly good at realizing efficient trades today may be awful at it tomorrow, and new entrepreneurs emerge, both in addition to the tumult that typifies the modern marketplace. However, for laboratory and field experiments conducted over short periods of time, experience is a good proxy for the abilities that we are attempting to capture. In the lab, to capture Hayekian entrepreneurship more directly, one would want to endow certain people with varying degrees of information about other market participants. Then one could see whether such insider knowledge leads to further rent generation for the market (as Hypothesis 2 claims) or to further rent extraction to the knowledgeable participant at the expense of the rest of the market.

### 3.5. Hypothesis 3: Price information and efficiency

Moving on to the next hypothesis, as we have already argued, in Hayek’s understanding of the competitive market process perfect knowledge cannot be viewed as a precondition for an equilibrium to emerge, but instead must be viewed as the defining characteristic of that equilibrium. The competitive process generates this knowledge as an outgrowth of the buying and abstaining from buying behavior of individuals within the economy. The optimality conditions of choice and of the system are results of the competitive process, and cannot be confused with assumptions of behavior and outcomes going in [32: p42].

Equilibrium corresponds to a situation where the plans of economic actors on both sides of the market are consistent with one another, “or, to put the same thing in less general and less exact but more concrete terms, that the expectations of the people and particularly of the entrepreneurs will become more and more correct.” [32: p45] But economics as an empirical science must be able to explain (a) the *conditions* under which the tendency to equilibrium exists, and (b) the *process* by which individual knowledge is acquired, utilized, and communicated so that the dovetailing of plans can occur.

In developing his theory of the market process and the theory of the discovery, use, and communication of knowledge it is important to stress that Hayek carefully distinguishes between full and complete knowledge of the system, and *relevant knowledge*. His theory works on the discovery and exploitation of the relevant knowledge of particular people located in a unique time and place. There is a *division of knowledge* in society, that must be coordinated just as the division of labor, to realize the gains from social cooperation. But too often in equilibrium economics, “instead of showing what bits of information the different persons must possess to bring about that result, we fall in effect back on the assumption that everybody knows everything and so evade any real solution of the problem.” [32: p51].

The relevant knowledge is revealed to individuals through the very process of exchange within the market economy as they attempt to better their circumstances. “We must look at the price system as such a mechanism for communicating information if we want to understand its real function … The most significant fact about this system is the economy of knowledge with which it operates, or how little the individual participants need to know in order to be able to take the right action. In abbreviated form, by a kind of symbol, only the most essential information is passed on and passed on only to those concerned.” [1: p86].

**Hypothesis 3 (Hayek [1, 35])**: Increasing the availability of information about trade prices has a positive causal effect on the realized proportion of efficient rents.

Except for *Hypothesis 3*, there are no testable predictions about prices. In contrast, in the conventional Walrasian model, since traders are assumed to be price-takers, the *only* price that guarantees the realization of first-best trades is the market-clearing price, and so the model makes a very specific, testable prediction about prices.

### 3.6. Hypothesis 4: Asymmetric information, reputation and efficiency

The final hypothesis relates to asymmetric information. Neoclassical economics started to formally analyze the consequences of asymmetric information in the 1970s. Early studies focused on demonstrating how asymmetric information could be a barrier to efficient trade [20]. Subsequent studies have explored institutions that can limit the damaging effects of asymmetric information [33], and the mechanism design literature uncovered clever contracts that could, under certain conditions, resecure first-best efficiency. Important contributions included the folk theorem and the reputational signaling literature [34], which established that reputational concerns could help overcome asymmetric information problems.

Hayek analyzed the causes and consequences of asymmetric information, though without the formal, game-theoretic tools of the 1970s and 1980s. As we have seen Hayek’s theory of the market process focuses on the division of knowledge in society, and as such postulates a universal condition of asymmetric information. “In actual life the fact that our inadequate knowledge of the available commodities or services,” Hayek wrote, “is made up for by our experience with the persons or firms supplying them–that competition is in large measure *competition for reputation or good will*–is one of the most important facts which enable us to solve our dialing problems.” [35: p97, emphasis added] Competition teaches us precisely who will best serve us within the market.

As Vernon Smith [25] has put it:

“If human agents with private and therefore inherently universal asymmetric information can converge to equilibrium outcomes by means that theorists can neither model nor predict, why would you believe that the market failure theorems derived from asymmetric information apply to those agents?”

Knowledge is dispersed but coordinated through the price system; competition disciplines through the profit and loss mechanism. Relevant knowledge is communicated to decision makers, and reputations are earned and lost based on the judgment of buyers within the market. Competition compels individuals to constantly “adjust their way of life” to adapt to the rapidly changing circumstances on which economic growth and development depends. (see [24: p189])

Smith further argues:

“We are left with Hayek’s critique [of equilibrium theory], his statement of the problem solved by decentralized pricing, and the experimental evidence supporting Hayekian efficiency in a wide variety of environments and institutions, but the theory showing how this works eludes articulation by means of the economist’s standard tool-kit.” [24: p107].

Following Smith, we want to see if field experiments find Hayekian efficiency to be as robust as has been found in the laboratory environment.

**Hypothesis 4 (Hayek [35])**: In markets with acute asymmetric information, introducing a reputation mechanism has a positive causal effect on the realized proportion of efficient rents.

### 3.7. Alternatives to the Hayek hypothesis

In addition to describing and testing the Hayek hypothesis, Smith [2] discussed two alternatives. The price-taking hypothesis [36, 37] is the foundation for Walrasian and neoclassical general equilibrium theory. In contrast to the Hayek hypothesis, it predicts efficiency if the number of traders is so large (infinitely large) that each trader has an imperceptible impact upon prices, while retaining the assumption of economy of information available to each agent. Modern bargaining models [38] relax the assumption of large numbers of agents at the expense of requiring ex ante complete knowledge of demand and supply [19]; Smith termed this the complete knowledge hypothesis. It is his dissatisfaction with both these classes of model that drove Smith towards testing the Hayek hypothesis.

## 4. Field experimental evidence on Hayek’s theories

Our operationalized versions of Hayek’s theories have been reduced to statements about the causal effect of features of the trading institution and trader attributes on the efficiency of resulting allocations. Smith [2] handled the laboratory experimental evidence. We here present the field experimental evidence.

### 4.1. Hypothesis 1: The Hayek hypothesis

The key field experiments are [11, 39]. In the first set of experiments described in [11], a variety of markets are considered where *N*_*B*_ = *N*_*S*_ = 12⇒*N* = 24, a small number, especially when compared to the asymptotic numbers called for in the Walrasian model. There are three market types: symmetric, shown in Fig 1, asymmetric demand elastic and asymmetric supply elastic.

In the symmetric markets, the distributions of values/costs imply that it is efficient for the 7 buyers who have a value strictly above $13 to trade with the 7 sellers who have a cost strictly below $14. Each trader is aware of his value/cost only. In the asymmetric demand (supply) elastic markets, supply (demand) was unchanged while demand (supply) was perfectly elastic at a price of $13.50, implying that it was efficient for any 7 of the 12 buyers (sellers) to trade.

The trading institution is decentralized multilateral bargaining (Chamberlin market) adapted to trading conventions for professional sports memorabilia. In real, non-experimental trading conventions, the role of sellers is taken by professional dealers, who rent tables in large exhibition halls and display their wares. These traders usually have extensive experience and knowledge about the value of the commodities both to themselves and more generally in the market. The role of buyers is mostly taken by amateur sports memorabilia enthusiasts who have substantially less experience than the dealers and attend the conventions as non-dealers. The buyers mill around, examining the dealers’ wares, bargaining within earshot of other traders and striking deals.

List virtually replicated this environment with a few modifications to permit the control necessary for inducing values/costs [11]. He recruited the participants inside an actual trading convention and ran it there. Recruited dealers were assigned the role of sellers and recruited non-dealers took the role of buyers; the sellers retained their real, rented desks and the buyers milled around (they had a map that clearly indicated the locations of the desks). The non-standard subject pool and the allocation of subjects to roles that mimicked their natural roles meant that this was a framed field experiment [5] in contrast to the laboratory experiments reviewed in [2]; see the discussion in Section 2 for the advantages that this conferred. Participants took part in five rounds of play in the same market, and the experiment lasted around 60 minutes.

The principal difference between the environment in [11] and a real trading convention is that a unique, homogenous commodity was being traded and its values/costs were induced in the standard manner used in laboratory market experiments (Smith 1976). Traders were told that the values/costs of others could differ, but no additional information about the distribution of values/costs was provided [11]. Was therefore a framed field experiment that satisfied conditions (a) to (e) in *Hypothesis 1*.

Table 1 contains the data’s principal features. Note that average prices are prices averaged across the multiple traders within a session and across sessions, whereas all other variables only have one observation per session and thus are averaged only across sessions.

**Table 1:** Main sample statistics from Experiment 1 in [11].

	Market period				
	1	2	3	4	5
	Symmetric markets				
Average price	13.5	13.9	13.7	13.8	13.1
Quantity	7.3	8	7	7	7.3
Efficiency	89%	85%	88%	87%	95%
Number of sessions	6	6	6	6	6
	Asymmetric demand elastic markets				
Average price	12.4	12.4	13.1	13.1	13.1
Quantity	5.7	6	6.6	7	6.3
Efficiency	87%	90%	99%	100%	90%
Number of sessions	6	6	6	6	6
	Asymmetric supply elastic markets				
Average price	15.9	16.1	14.4	13.9	13.7
Quantity	3.6	4.3	6.3	7	6.3
Efficiency	73%	80%	96%	100%	96%
Number of sessions	6	6	6	6	6

The simple average of efficiency in the 90 sessions in Table 1 is 90%, rising to 95% if we focus purely on the 54 sessions in market periods 3-to-5.

[11] also conducted similar experiments but with children under the age of 13 randomly assigned to the role of buyer or seller. Sessions used one of two demand and supply systems: 24-children sessions used the system in Fig 1, while 12-children sessions used a condensed version that required 4 trades for full efficiency. The 24-children sessions were always run with children with randomly varying real trading experience. There were three types of 12-children sessions: those with children whose experience varied randomly, those with experienced children and those with inexperienced children. Experienced children have traded more than 12 times per month in the last two years. Inexperienced children have traded less than one time per month in the last two years. Similar to the adult sessions, all children gained substantial experience *during* the experiment by participating in five market periods.

The main sample statistics are in Table 2. Average efficiency across all sessions and periods is 69%, which is substantially lower than for adults, though still large compared to the 0 efficiency of autarky. For (naturally) experienced children only, efficiency is 89%, and across all sessions but only for the last two periods, efficiency is 87%.

**Table 2:** Sample statistics for children from Experiment 1 in [11].

	Market period				
	1	2	3	4	5
	Symmetric markets: 24 random children				
Quantity	3	3.7	6	6.3	7.7
Efficiency	43%	40%	71%	84%	95%
Number of sessions	6	6	6	6	6
	Symmetric markets: 12 random children				
Quantity	2	3	4	4	3
Efficiency	59%	18%	91%	100%	97%
Number of sessions	2	2	2	2	2
	Symmetric markets: 12 experienced children				
Quantity	3	4	3	4	3
Efficiency	68%	100%	82%	100%	97%
Number of sessions	2	2	2	2	2
	Symmetric markets: 12 inexperienced children				
Quantity	1	3	3	3	2
Efficiency	35%	62%	47%	29%	71%
Number of sessions	2	2	2	2	2

These data are complemented by regressions on the adults’ experiments that control for the effect of a variety of naturally-occurring and experimentally-manipulated regressors; [11] found that more experienced traders (on both sides of the markets) earned more than their less-experienced counterparts. Together, these data are consistent with Hayek’s claim that a crucial component of the market mechanism was the natural selection it imposed upon traders: poor traders would be eliminated, and superior traders would survive (and thrive) [1].

To explore the robustness of his results from sports memorabilia trading conventions [11], collected data in a market for a different type of collectible (collector pins). This latter market was dominated by women, unlike the male-dominated sports memorabilia markets. Using the 12-trader versions of systems in Fig 1, List found efficiency levels that were virtually identical to those in the preceding experiments.

[39] extend the design in [11] with three new sessions using the same demand and supply system as in Fig 1, but with four sellers each selling three units (rather than 12 sellers each selling one unit). All other features are identical. The resulting efficiency levels for periods 1-to-5 were 95%, 98%, 96%, 98% and 94% respectively, lending further support to the data in [11]. (They also ran sessions where they facilitated collusion; we discuss these below.)

**Result 1**: A large proportion of efficient rents are realized even if the five conditions in *Hypothesis 1* hold.

In a follow-up paper based on the data from [11, 39, 40] investigate the source of the small efficiency losses observed in the Chamberlin markets. Using a combination of simulation and empirical analysis, they conclude that some of the efficiency losses are due to relationships forming between traders even in the presence of perfect external enforcement of trading contracts. These relationships, which they argue reflect preferences for bargaining with people who have a similar bargaining style, increase the likelihood that an extramarginal trader secures a trade with an intramarginal trader.

*Result 1* is consistent with the laboratory data synthesized by Smith [2]. While collecting data from field experiments has not changed the conclusion emerging from laboratory studies, it has made us more confident in their generalizability. For example, the cited field experiments demonstrate that the laboratory results are robust to the presence of participants with years of experience, and such experience levels are common in many naturally-occurring markets. In List’s experiments in sports memorabilia conventions average trader experience for dealers is around 10 years with a standard deviation of around 7 years, and for non-dealers is around 7 years with a standard deviation of around 10 years. Moreover, allowing participants to self-select into their natural roles does not alter *Result 1*. In both cases, there are examples in other fields where results are sensitive to such differences (see [6]), and so there is always value in running field experiments to explore robustness.

### 4.2. Hypothesis 2: Entrepreneurs and efficiency

[41] virtually replicates the experimental design in [11] but systematically varies the experience-level of the traders on each side of the market in markets populated by adults. List ran four versions of the 24-trader market in Fig 1: (1) random levels of experience for all traders, (2) experienced buyers with inexperienced sellers, (3) experienced buyers with experienced sellers, and (4) inexperienced buyers with experienced sellers. The results are in Table 3. Unlike [11] the sample is quite small: List only ran one session of each version in Table 3, and so the results should be interpreted cautiously.

**Table 3:** Sample statistics from [41].

	Market period				
	1	2	3	4	5
	Random				
Quantity	8	9	7	7	7
Efficiency	89%	86%	86%	97%	97%
	Experienced buyers/Inexperienced sellers				
Quantity	7	8	7	6	8
Efficiency	84%	97%	97%	95%	95%
	Experienced buyers/Experienced sellers				
Quantity	8	7	7	9	7
Efficiency	86%	80%	81%	78%	95%
	Inexperienced buyers/Experienced sellers				
Quantity	6	7	6	7	7
Efficiency	95%	81%	95%	59%	97%

Somewhat surprisingly, the prevalence of entrepreneurs seems to have a non-monotonic effect on the realization of rents. Efficiency is highest when buyers are experienced and sellers inexperienced (93%), and lowest when both are experienced (82%). Estimating rents as a function of the presence of experienced traders at the level of the individual (thereby creating 24 observations per session) confirms that this difference is statistically significant.

[39] provides evidence in support of *Hypothesis 2*, but the data are more difficult to interpret. In addition to the 12 single-unit buyer, 4 3-unit seller markets described above, they also ran sessions where the 4 sellers were allowed to meet privately and collude. In some of the sessions, sellers clearly succeeded in collusively improving their rents at the expense of global efficiency. The authors did not explicitly manipulate the experience level of the buyers; however, they found that when at least one unit was purchased by an experienced buyer, some of the efficiency losses from collusive sellers were reversed.

[42] find indirect support for *Hypothesis 2*. Although they did not directly vary experience, they studied “subjects from rural African villages with very little access to markets or experience in trading” (p315). These inexperienced traders realized lower gains from trade and markets did not converge, despite using double-sided oral auctions as in [11].

So far, we have only considered double auctions, i.e., where both buyers and sellers can offer trade prices [43]. Conduct field experiments in a single auction environment. Their goal is to study the incidence of the winner’s curse (WC): in common-value auctions with imperfect information and private signals above the common value, the tendency for participants to overbid.

Harrison and List’s [43] first study is an artefactual field experiment. They recruit dealers and non-dealers from the floor of a sports memorabilia convention; the total number of participants in each auction *N* is either 4 or 7. There is an item whose value *v* is drawn randomly from the range [$40,$200]; each of the bidders receives a private signal of the value distributed uniformly in the range [*v*−*ε*, *v*+*ε*], where *ε* was either $6 or $12 depending on the treatment. In symmetric sessions, all bidders received an IID private signal; in asymmetric sessions, all but one received an IID private signal, and the last bidder was an insider who knew the exact common value. All these facts were common knowledge.

Ex post efficiency requires that the item be transferred to the highest bidder if and only if the bidder’s value exceeds the seller’s value (the value is common across bidders only). If the winning bid is always (weakly) less than the bidder’s value, then it is impossible for the bidder to inefficiently purchase the good. A necessary condition for a potentially inefficient purchase by the bidder is that the bidder bid above his value, and this is the principal efficiency concern underlying the WC. Thus, the more likely are bidders to bid above the actual common value, the more likely an inefficient allocation.

In these experiments, the common value was always $94.33. Harrison and List found that in all treatments, bidders show a non-trivial tendency to bid in excess of $94.33, however the dealers, who represent the entrepreneurs, are less likely to suffer from this potential source of inefficiency. The main results are in Table 4. The percentages are approximate since they are retrieved from eye-balling data displayed in bar charts. In the columns for the artefactual field experiment, we can see that in all treatments, dealers on average bid lower than do non-dealers (these differences are statistically significantly different from zero). Moreover, dealers are across-the-board less likely to bid in excess of the WC threshold.

**Table 4:** Sample statistics from [43].

Information treatment		Artefactual		Framed
		*N* = 4	*N* = 7	*N = 4*
Symmetric	Mean dealer bid—Mean non-dealer bid	-$2.23	-$2.40	-$1.21
	% of dealer bids exceeding WC threshold	5%	7%	-
	% of non-dealer bids exceeding WC threshold	38%	47%	-
	Observations	96	98	64
Asymmetric	Mean dealer bid—Mean non-dealer bid	-$3.50	-$3.92	-$2.31
	% of dealer bids exceeding WC threshold	32%	26%	-
	% of non-dealer bids exceeding WC threshold	81%	79%	-
	Observations	112	49	60

The second study was a framed field experiment with an analogous setup, but where the item to be auctioned was a pack of sports cards that potentially contained some valuable cards, implying that the item’s value was homegrown rather than induced. As can be seen in the last column of Table 4, dealers again bid less than non-dealers on average, though since homegrown values are unobservable, it is impossible to definitively claim that the non-dealers were overbidding.

[44] study how imperfect information can hamper the efficiency of financial markets. The true state of the world is *ω*∈{*A*, *B*}. A set of agents *I* = {1,2,…,*n*} does not know the state of the world, but each receives an independent signal *s*_*i*_∈{*a*, *b*} about the state where Pr(*A*|*a*)>Pr(*B*|*a*) and Pr(*A*|*b*)<Pr(*B*|*b*). The first agent sees his signal and chooses *c*_1_∈{*A*, *B*}, his guess about the true state of the world; the second agent sees his own signal and *c*_1_, and then makes his guess, *c*_2_. Each agent sees the choices of *all* preceding agents, in addition to his own signal, before making his choice. After all agents have guessed the state of the world, the true state is revealed; agents who guessed correctly (*c*_*i*_ = *ω*) earn one unit; those who guessed incorrectly earn zero.

The actual probabilities, which are common knowledge, imply that cascades can form. A cascade occurs when an agent’s rational guess (in the Bayesian sense) about the state of the world is based purely on the history of guesses and is insensitive to his private signal. When a cascade commences, the stock of useful information based on the agents’ private signals and embodied in their decisions ceases to grow, despite the fact that a large number of agents could correctly guess the state of the world with near certainty if they pooled their private information. From an efficiency viewpoint, of particular concern is the possibility of a *reverse cascade*, whereby agents rationally herd on the incorrect state.

Generalizations of the model presented in [44] are widely applied to markets for assets with uncertain payoffs. Firms issue assets in exchange for investors’ capital. The main question is if the imperfect information impedes the markets’ ability to allocate capital to investment projects efficiently. The literature on cascades in financial markets has been used to argue against the Hayek hypothesis, since reverse cascades constitute failures to realize efficient outcomes.

[44] run a laboratory experiment and a framed field experiment in an environment described by the above model. Participants are recruited and play 15 rounds in groups of five or six. In the laboratory version participants are college students; in the framed field version the participants are professional traders from the Chicago Board of Trade. The professional traders correspond to Hayekian entrepreneurs since arbitrage is their primary activity.

The authors vary the probabilities across sessions to explore the determinants of any cascades, though this is not directly related to *Hypothesis 2*. They also vary the frame between gains and losses.

Table 5 contains the main data on reverse cascades. In aggregate, reverse cascades are quite infrequent, occurring 3.3%. However, when we differentiate between the markets populated by Hayekian entrepreneurs (market professionals) and those composed of non-entrepreneurs (college students), we can see that the Hayekian entrepreneurs generate less than half the proportion of reverse cascades (this difference is significant at the 5% level). In results that we will discuss more fully below, market professionals’ behavior–unlike that of college students–is unaffected by payoffs being framed as gains vs. losses.

**Table 5:** Sample statistics from [44].

Participants	Observations	Reverse cascades
College students	822	37 (4.5%)
Market professionals	825	18 (2.1%)

The literature on the endowment effect offers important data on the causal effect of Hayekian entrepreneurs on efficiency. The endowment effect is defined as a large discrepancy between the amount an individual is willing to pay to acquire a good and the amount he must be paid to relinquish the same good once it has come into his possession (the latter exceeding the former). It is ‘large’ in the sense that it cannot be plausibly explained by income effects, and that it is a consequence of loss-aversion [45] and/or the human affinity for physical possessions once they have been acquired. For example, people tend to become very fond of mass-produced, cheap pens once they have used them.

In the classic Walrasian markets model, a trader’s final allocation under the competitive (and efficient) equilibrium is not affected by variations in his endowment conditional on the value of his endowment at the equilibrium price vector. The price mechanism still guarantees that goods will flow to those who value them the most. This process is impeded by the endowment effect; if initial endowments are at least partially arbitrary, then the endowment effect acts as an inefficient transaction cost. It makes traders who ‘ought’ to sell their good reluctant to do so, limiting the realization of efficient rents (see [41] for a discussion).

Consequently, if the endowment effect is somewhat prevalent and Hayekian entrepreneurs suffer less from it than do non-entrepreneurial traders, then this would constitute evidence in favor of *Hypothesis 2*. Several field experiments on the endowment effect provide such data.

[41] invites dealers and non-dealers at sports memorabilia conventions to participate in a short survey in exchange for a piece of sports memorabilia. List chose two approximately equally valued goods: A (a ticket stub from a famous baseball game) and B (a certificate commemorating a momentous achievement by a famous baseball player). People who agree to participate in the survey experience the following:

1. They are physically handed one of the two goods (chosen at random)
2. They fill out the survey (which takes around five minutes)
3. The remaining good is offered to them in exchange for the first good, and they decide if they want to trade or not
4. They complete a short exit survey

There were several noteworthy features of the design. First, the participants had the good in their physical possession while they filled out the survey, which has been shown to enhance the endowment effect. Second, pre-testing ensured that the two goods were of equal value. Third, the goods were chosen on the basis that they would be consumed rather than sold elsewhere, and the exit interview confirmed that this was the case for 95% of the participants.

The results are in Table 6. In the pooled data, List found data consistent with previous demonstrations of the endowment effect: the sum of the percentages of those who traded A for B and B for A is 68%, which is significantly less than 100%. However, when List broke the sample down into dealers and non-dealers, he found that the endowment effect was almost exclusively driven by non-dealers: the sum of the two percentages was 89% for dealers (not significantly different from 100%) and 46% for non-dealers (significantly less than 100%).

**Table 6:** Sample statistics from Experiment 1 in [41].

Sample	Variable	Percent traded
Pooled (n = 148)	Received A and traded for B	32.8
	Received B and traded for A	34.6
Dealers (n = 74)	Received A and traded for B	45.7
	Received B and traded for A	43.6
Non-dealers (n = 74)	Received A and traded for B	20.0
	Received B and traded for A	25.6

Additional robustness tests confirmed the basic result. Thus, for example, dividing the sample of non-dealers into experienced- and inexperienced dealers reveals that experienced non-dealers are substantially less likely to suffer from the endowment effect. Controlling for observable attributes in a regression, such as demographics, does not alter the main result. List also conducted an analogous experiment in a pin-trading market and found similar results. Using a similar environment [46], also finds evidence that experienced traders are less likely to suffer from the endowment effect. Finally, as mentioned above [44, 47], and found that professional traders playing Bayesian signal-extraction games were less likely to suffer from loss-aversion.

In contrast to [41, 44, 46–48] finds that professional traders are more likely to suffer from myopic loss aversion than college students, and that they suffer from it quite acutely. They employ the design of [49]: in each round of a game, participants are asked to decide how many of 100 units to invest in a risky project that returns 2.5 units per unit invested with probability 1/3 and nothing with probability 2/3. This game is played for nine rounds.

In the *frequent* treatment, each participant receives feedback about his earnings at the end of each round and before making his choice for the next round. In the *infrequent* treatment, participants made their choices for three consecutive rounds and received feedback only about their aggregate earnings and only at the end of the three rounds. Thus, the payoff structure was identical across treatments, but a loss is more likely to be perceived in the frequent treatment because feedback is more frequent. Myopically loss averse individuals should invest more in the infrequent treatment. Similar to the endowment effect, this cognitive bias impedes efficient trade.

[48] run the experiments on a group of college students and a group of professional traders from the Chicago Board of Trade (an artefactual field experiment). They find that both college students and professional traders suffer from myopic loss aversion. They also find that professional traders invested less than college students in the frequent treatment and more than them in the infrequent treatment, rendering them more acute sufferers of myopic loss aversion. Unfortunately, the authors do not attempt to reconcile this finding with the more common finding that professionals are less likely to suffer from behavioral anomalies (see [50]).

**Result 2**: Generally speaking, entrepreneurs have a positive causal effect on the realized proportion of efficient rents, though there are some exceptions.

Similar to *Result 1*, *Result 2* is supported by a wealth of laboratory data, e.g., for analyses of the role of experience, see [51, 52], but this again does not undermine the usefulness of complementing the laboratory evidence with field experiments in light of the reasons for plausibly expecting differences [6]. Moreover, field data allows one to dig deeper into the causal mechanisms, e.g., using a structural model [41], compares treatment and selection as reasons why traders in a specific, naturally-occurring markets suffer less from the endowment effect.

### 4.3. Hypothesis 3: Price information and efficiency

Recall that in [39], the authors ran Chamberlin markets with 4 sellers each selling 3 units, and 12 buyers each buying 1 unit (the system is depicted in Fig 1). This was a framed field experiment because the sellers were dealers and the buyers were non-dealers, all recruited from sports memorabilia conventions. In a subset of sessions, the sellers were allowed to privately collude for a few minutes prior to the start of trading. These sessions were divided into two types: perfect information sessions, where after each trade the transaction price was declared publicly; and imperfect information sessions, where after each trade the transaction price *plus a uniformly distributed white noise term* was declared publicly. The information structure was common knowledge. Thus, the perfect information sessions correspond to a greater availability of information about trade prices.

Table 7 contains the main results. Average efficiency is 86% in the perfect information sessions and 91% in the imperfect information sessions. Using a Mann-Whitney test, this difference is marginally significant (p < 7%), and it contradicts *Hypothesis 3*. The explanation is that when price information is transmitted perfectly, it is relatively straightforward for sellers to monitor other sellers and enforce the terms of a cartel, which are inefficiently high prices.

**Table 7:** Sample statistics from [39].

	Market period				
	1	2	3	4	5
	Perfect information (3 sessions)				
Quantity	4.7	4.3	5	4.7	5
Efficiency	84%	84%	86%	86%	90%
	Imperfect information (3 sessions)				
Quantity	5.3	5.7	5.7	6.3	5.7
Efficiency	97%	92%	84%	91%	92%

In additional sessions where the buyers were experienced [39], found that both treatment and control delivered (approximately) efficient outcomes.

To the best of our knowledge, this is the only field experiment in which an investigator has experimentally manipulated the dissemination of price information. Since the context is very narrow, we are cautious in generalizing the conclusion to other, more frequently occurring contexts (such as those considered in [11]).

**Result 3**: There is some evidence that increasing the availability of information about trade prices has a negative causal effect on the realized proportion of efficient rents.

Laboratory experiments have been employed to study similar questions, with similar conclusions [53, 54]. The principle difference between the framed field experiment in [39] and the aforementioned laboratory studies is the natural, endogenous selection in roles, which the authors expected to potentially yield different results. A novel result in [39] was the sensitivity of the effect of more accurate price information to the presence of experienced buyers, though admittedly this can also be tested in a laboratory setting. Regardless, as with previous results in this paper, the field permitted the researchers to observe how experience at the level of years affected conclusions.

### 4.4. Hypothesis 4: Asymmetric information, reputation and efficiency

Experimentally modifying the extent of reputational concerns in a naturally-occurring market is difficult [55]. Designs a field experiment around a fortuitously timed natural experiment to generate data relevant to this *Hypothesis 4*.

[55] builds on the laboratory gift exchange literature [56], which is similar in structure to the trust game [57] and is essentially a sequential prisoner’s dilemma. A sender and responder participate in a one-shot interaction. The sender sends an amount *x*∈[0, *a*] to the responder, retaining (*a*−*x*) for himself. The amount *x* is tripled, disclosed to the receiver and then the receiver unilaterally chooses how much to return to the sender, *y*_*x*_∈[0,3*x*], keeping the remaining (3*x*−*y*) for himself. The final payoffs are (*a*−*x*+*y*_*x*_, 3*x*−*y*_*x*_).

Efficiency requires *x* = *a*, while the unique subgame Nash equilibrium is (*x* = 0, *y*_*x*_ = 0 ∀ *x*). This game is considered an allegory for various market transactions with asymmetric information, such as worker effort under incomplete labor contracts [56] or product quality in markets with unobservable quality [20]. The responder takes the role of the party with private information, and the opportunity to exploit that information can prevent the realization of efficient exchange. Despite a vigorous debate over the interpretation of laboratory results of gift exchange games (see [6]), to the best of our knowledge [55], was the first field experimental test (presumably because it is difficult to locate an environment genuinely devoid of reputational concerns).

List’s design is again based on sports memorabilia markets. Subjects are instructed to approach dealers and offer them a low fixed amount or a large fixed amount for a piece of sports memorabilia of a certain quality. To an inexperienced buyer, quality is difficult to measure, and so the seller has an opportunity to ‘defect’ and deliver low quality. If ex post quality is sufficiently low and unaffected by the offer price, then this will dissuade buyers from initiating a purchase and will result in a failure to realize efficient trades.

List interacts the primary treatment variable (offered price) with two other explanatory variables. The first is whether or not the approached dealers are local, which is a sufficient condition for the existence of reputational concerns: local dealers frequently return to the same conventions and deal with repeat customers.

The second is the existence of third-party quality certification. For one of the items that List instructed subjects to purchase, in the early stages of the experiment there was no third-party quality certification. In the experiment’s latter stages, a third-party quality-certifier emerged. The (un)availability of quality certification was common knowledge.

List finds that when third-party quality certification is available, pooled data for dealers exhibits quality that is price-contingent. However, upon distinguishing between local and non-local dealers, the positive relationship exists only for local dealers. In contrast, in the period where quality certification is absent, all dealers deliver low quality that is insensitive to offered price.

These results both support and fail to support *Hypothesis 4*. The introduction of a reputation mechanism (the third-party quality certifier) only aided the realization of efficient trades for local traders. For non-local traders, the reputational mechanism was too weak to overcome agency problems because of the projected infrequency of future interactions in the market.

To the best of our knowledge [54], is the only field experiment that studies the effect of a quasi-exogenous shock to the existence of a reputation mechanism [58, 59]. Both study eBay auctions and demonstrate that the reputation mechanism in eBay (buyers rate sellers after transactions) works in the sense that sellers with better reputations deliver higher quality products. However, they do not present evidence of how the *introduction* of a reputation mechanism actually affects the realization of efficient trades [60]. Examine the effects of the introduction of professional certifiers into the sports card market on grading, and they also study the nature of competition between the incumbent and entering certifiers. However, their results do not provide any direct evidence of efficiency improvements arising from the certification [61]. Study group membership and identity, which are related to reputation. However, they study how group membership helps a group of sellers collude to raise prices and lower efficiency and ignore the interaction between reputation and asymmetric information.

**Result 4**: There is some evidence that in markets with acute asymmetric information, introducing a reputation mechanism has a positive causal effect on the realized proportion of efficient rents.

Laboratory experiments have long been used to study the ability of reputational concerns to overcome inefficiencies arising from asymmetric information, starting with repeated prisoner dilemmas. In the context of bilateral transactions, there are good reasons to expect social preferences to affect the interaction [55], but laboratory experiments suffer from elevated scrutiny that potentially bias results [6]. This makes natural field experiments such as [54] an obvious complement to the laboratory evidence.

In this sense, the novel (compared to laboratory experiments) result in [54] is the uncovering of a naturally occurring reputational mechanism that fails to overcome asymmetric information-induced inefficiencies. Moreover, the use of a field setting allowed List to consider the effect of reputational horizons of years (in the case of local dealers), which would be otherwise difficult to induce in a laboratory setting.

## 5. Conclusion

Viewed through Vernon Smith’s lens, Hayek’s theories yield multiple testable hypotheses, the most important of which is the ability of the market to deliver efficient outcomes despite price-setting, the presence of rampant, imperfect/incomplete information and the absence of central market direction. Smith examined the laboratory evidence and found strong support for the Hayek hypothesis.

Market field experiments are a complementary source of evidence that did not exist at the time of Smith’s review. A key advantage of field experiments is that they allow us richer tests of Hayek’s hypotheses. For example, when using experience as a proxy for entrepreneurship, the range of experience that can be induced and/or obtained in a standard laboratory experiment is relatively small. In a framed field experiment, one can observe differences in experience of years or thousands of trades. Similarly, for reputational concerns to affect trading behavior, it is possible that horizons of months and years are necessary, and this may be logistically easier to obtain in a field experiment. Certainly, we know that real markets do have people with years of experience and long reputational horizons interacting, and so reproducing that in a (framed) field experiment is a sensible departure point. Whether field experiments and laboratory experiments with lower experience levels/shorter horizons produce similar results is an open empirical question. Likely the best insights are obtained by combining the two sources of evidence and exploiting the advantages of each.

We find that in general, market field experiments support Hayek’s theories. Most significantly, and reassuringly given Smith’s findings, the Hayek hypothesis seems as robust in the field as it is in the laboratory. We also find multiple dimensions of evidence supporting Hayek’s proposition that entrepreneurs are a key force in driving markets to equilibrium and in realizing the gains from trade.

There is a rich tradition among neoclassical economists of studying price dynamics and the market process. Somewhat surprisingly, the theoretical literature of the last 30 years has paid little attention to Hayek’s theories, though undoubtedly this is at least partially a consequence of the difficulty of tractably formalizing them. Nevertheless, we hope that in concert with Smith, this study encourages theorists to re-examine Hayek’s theories.

We also find that some of the field experimental data is less supportive of Hayek’s theories. There is evidence that reputation cannot alone solve problems of asymmetric information, and the (admittedly very narrow) evidence on the effect of disseminating price information poses questions of Hayek’s claim that it promotes efficient trade.

In the case of the more weakly supported theories, are we to conclude that Hayek was simply wrong? Can his theories be refined in response to the findings? Perhaps the data presented are poor tests of Hayek’s theories, or they are unrepresentative of market processes in general. We leave these questions to future research.
